# Red Queen dancing in the lek: effects of mating skew on host–parasite interactions

**DOI:** 10.1002/ece3.1809

**Published:** 2015-11-02

**Authors:** Kazutaka Kawatsu

**Affiliations:** ^1^ Department of Environmental Solution Technology Faculty of Science and Technology Ryukoku University 1‐5 Yokotani Seta Oe‐cho Otsu Shiga 520‐2194 Japan

**Keywords:** Female choice, host–parasite coevolution, individual‐based model, maintenance of sex, male–male competition, Red Queen hypothesis, sexual selection

## Abstract

The RQH (Red Queen hypothesis), which argues that hosts need to be continuously finding new ways to avoid parasites that are able to infect common host genotypes, has been at the center of discussions on the maintenance of sex. This is because diversity is favored under the host–parasite coevolution based on negative frequency‐dependent selection, and sexual reproduction is a mechanism that generates genetic diversity in the host population. Together with parasite infections, sexual organisms are usually under sexual selection, which leads to mating skew or mating success biased toward males with a particular phenotype. Thus, strong mating skew would affect genetic variance in a population and should affect the benefit of the RQH. However, most models have investigated the RQH under a random mating system and not under mating skew. In this study, I show that sexual selection and the resultant mating skew may increase parasite load in the hosts. An IBM (individual‐based model), which included host–parasite interactions and sexual selection among hosts, demonstrates that mating skew influenced parasite infection in the hosts under various conditions. Moreover, the IBM showed that the mating skew evolves easily in cases of male–male competition and female mate choice, even though it imposes an increased risk of parasite infection on the hosts. These findings indicated that whether the RQH favored sexual reproduction depended on the condition of mating skew. That is, consideration of the host mating system would provide further understanding of conditions in which the RQH favors sexual reproduction in real organisms.

## Introduction

Infectious disease and parasite load are pervasive in nature and impose a heavy cost on host species (Poulin 2007; Schmid‐Hempel 2011). Given that host resistance against infection has a genetic basis, parasites with a short generation time will be able to adapt rapidly to common host genotypes, favoring rare hosts. As a result, this host–parasite coevolution generates negative frequency‐dependent selection, which favors mechanisms that increase the genotypic diversity of the host populations. This is known as the “RQH” (Red Queen hypothesis, Van Valen 1973), which has been at the center of the debate in studies on the evolution of sex because sexual reproduction involves recombination or genetic mixing that produces offspring with various genotypes (Bell 1982). Many theoretical studies have revealed conditions in which the RQH favors sex and recombination (e.g., Jaenike 1978; Hamilton 1980; Hamilton et al. 1990); however, those studies have focused mainly on the RQH under random rather than nonrandom mating systems of host species.

In general, the maintenance of sex becomes problematic under the assumption that paternal investment in the offspring is minimal (Kawatsu 2013a,b), as producing male offspring is completely wasteful in terms of population growth (the twofold cost of sex; Maynard Smith 1978). In addition, the minimal paternal effort is a key force driving other evolutionary outcomes: Sexual difference in paternal investment (anisogamy) yields sexual selection, in which the two sexes undergo different selection pressures (Andersson 1994). Sexual selection leads to conditions of mating skew, which is typically seen as a biased mating success toward males with a particular phenotype (Andersson 1994; Keller and Reeve 1994; Kokko and Lindström 1997). For some genetic factors under a sexually selected trait, strong mating skew would affect genetic variance in the population (Gavrilets 2004). The benefit of sex with the RQH depends on the rate at which outcrossing produces genotypic diversity against the counter evolution of parasites (Otto and Nuismer 2004; Neiman and Koskella 2009). Therefore, sexual selection should consequently alter the Red Queen dynamics of host–parasite coevolution.

Recently, theoretical studies have shown that the RQH may promote the evolution of female mate choice for male disease resistance (Howard and Lively 2003, 2004; Nuismer et al. 2008), which leads to mating skew. In particular, the allelic frequency of female preference changes by associating with immunity genotypes that are favored or disfavored by host–parasite interaction (Nuismer et al. 2008). Although such findings imply that sexual selection can have positive and negative effects on the health of the host population, the effect of mating skew on the infection dynamics remains poorly understood. This is because previous studies addressed only the “evolution” of female preference; however, a broad range of factors other than female mate choice can cause mating skew. For example, male–male competition will increase the mating success of males with competent traits, and disease resistance will be associated with the trait if parasite infections are detrimental to health and the competence of host males (David and Heeb 2009). Even in the case of female mate choice, this would evolve whenever selection of a mating partner provided direct and/or indirect benefits to choosy females (Kokko et al. 2003). In fact, the benefits stem from many mechanisms, ranging from a heritable attractive male trait (sexy son, e.g., Fisher 1930; Lande 1980; Kirkpatrick 1982) to direct avoidance of infected partners (e.g., Borgia 1986; Ehman and Scott 2001, 2002; Kavaliers et al. 2005). Furthermore, although sexual traits do not at all indicate male immunity, sexual selection may impact the disease resistance of a host population because mating skew will amplify genetic drift or stochastic changes in the frequency of alleles in a finite population.

In this paper, I hypothesized that mating skew alters the Red Queen dynamics of host–parasite arms race and that sexual selection evolves mating skew even if it increases parasite load in the host population. To verify this hypothesis, an IBM (individual‐based model) was developed, which included host–parasite interactions and sexual selection in the host population, and the RQH was examined under different degrees of mating skew and genetic correlations between the health of the males and the male trait that was under sexual selection. Based on the results, the possibility of the evolution of mating skew with varying genetic correlations between the male traits and health was examined. In a second analysis, to investigate whether the evolution of mating skew in the host species differed between types of sexual selection; that is, male–male competition for mates and female mate choice, the IBM was extended to describe both a case in which mating bias was associated only with male–male competition and a case in which female preference for male traits also affected the evolution of mating bias. Discussion of these analyses should shed light on the demographic effects and evolutionary outcomes of mating skew in host organisms coevolving with parasites.

## Materials and Methods

An IBM that tracked the coevolutionary dynamics between hosts and parasites under sexual selection for host males was constructed. In the model, it was assumed that selection acted on haploid organisms, and the host and parasite population consisted of a constant number of individuals, *N*_H_* and *N*_P_*_,_ respectively. Hosts were assumed to be obligatory sexual and anisogamous species (i.e., characterized as male and female sexes), whereas parasites reproduced asexually. The model consisted of a two‐step selection phase: a host–parasite interaction and a mating interaction in the hosts. The host–parasite interaction was mediated by a haplotype of host immunity and parasite infectivity. Following the interspecific interaction, host mating proceeded; each host female was modeled to mate with one male depending on his genetic quality.

### Host–parasite interaction

The model assumed that outcomes of the interspecific interaction between a host and a parasite (i.e., infection or resistance) were mediated by a mechanism of self‐ versus non‐self‐recognition that was controlled by a haplotype of host immunity and parasite infectivity. Both haplotypes were simply determined by two loci with two alleles, and each allele was labeled either 0 or 1 (this assumption of genetics was for analysis convenience, and preliminary simulations of multilocus and/or diploid system suggested that the conclusions presented in this paper are qualitatively robust). For the recognition mechanism, host immunity could recognize parasites with surface molecules different from those of the host cell (Frank 2002) and effectively exclude them from the host body. This type of host–parasite recognition has been considered a possible immune function of animals (Frank 2002), and several genetic models of recognition mechanism have been used in theoretical studies on the Red Queen dynamics (e.g., Hamilton et al. 1990; Otto and Nuismer 2004; reviewed in Salathè et al. 2008). Specifically, MA (matching‐allele) genetics was applied to the recognition mechanism in this paper (i.e., a parasite infection occurred when an allele at an infectivity locus matched that of the corresponding locus of host immunity). The overall probability of infection was additively determined as the mean number of matching alleles between a host and a parasite (i.e., the model did not consider other biologically plausible genetics, such as a case in which each locus multiplicatively contributes the outcome of the infection).

In the model, it was assumed that each host individual with the immunity haplotypes *G*_*H*,*i*_ was randomly exposed to parasites with the infectivity haplotypes *G*_*P*,*j*_ depending on their frequency and that interactions resulting in infection reduced host fitness (Nuismer et al. 2008). Thus, the survival rate of a host with haplotype *G*_*H*,*i*_ could be represented as:

(1) $$S\left(G_{H,i}\right)=1-\theta_{H}\sum_{j}P_{j}\psi \left[G_{H,i},G_{P,j}\right],$$

where *ψ*[*G*_*H*,*i*_, *G*_*P*,*j*_] represents the probability that a host with *G*_*H*,*i*_ is infected by parasites with *G*_*P*,*j*_, whose frequency is denoted by *P*_*j*_. The parameter *θ*_*H*_ scales the strength of selection exerted by parasite infection. For parasites, their survival rate with haplotype *G*_*P*,*j*_ improves, contrary to the host survival rate, as the average probability of successful infection increases:

(2) $$S\left(G_{P,i}\right)=1-\theta_{P}\sum_{j}H_{j}\left(1-\psi \left[G_{H,j},G_{P,i}\right]\right),$$

where the parameter *θ*_*P*_ represents the scale of selection by host resistance, and *H*_*j*_ indicates the frequency of hosts with haplotype *G*_*H*,*j*_. To incorporate the effect of a short parasite generation time, it was assumed that parasites produced several generations per one host generation: The infection rate of the host is determined by the haplotype frequencies for parasite infectivity at the last parasite generation in a single host generation.

After interactions between the two species, each host and parasite was randomly selected to form a reproductive population according to their survival rates. In addition, to maintain the size of the reproductive population of the host and parasite below *N*_H_* and *N*_P_*, respectively, a density‐dependent mortality was imposed on hosts and parasites. Denoting the number of host and parasite individuals after host–parasite interaction by *N*_*H*_ and *N*_*P*_ at a generation, the density‐dependent mortality of hosts and parasite becomes max(0, 1 – *N*_H_*/*N*_*H*_) and max(0, 1 – *N*_P_*/*N*_*P*_), respectively.

### Host mating interaction

The model addressed a situation in which each host female mated with a male depending on his value on a sexually selected phenotype, *Y*. An expression of *Y* was associated with a continuous value of an allele of the male *y* trait and his health or state of infection to some degree; that is, the male phenotype *Y* could be referred to as a condition‐dependent sexually selected trait (Andersson 1986; Zeh and Zeh 1988; Iwasa et al. 1991). In the model, the following equation was used to determine the *Y* phenotype of a male with a *y* allele and *G*_*H*,*I*_ genotype:

(3) $$Y\left(y,G_{H,i}\right)=\left(1-\rho \right)y-\rho \left(1-\sum_{j}P_{j}\psi \left[G_{H,i},G_{P,j}\right]\right),$$

where the parameter *ρ* denotes the degree of correlation between the male phenotype and health. For example, when *ρ *= 0.0, only the value of the male trait determines the male phenotypes, whereas only the state of infection determines the phenotypic value when *ρ *= 1.0.

The first simulation investigated the effect of mating skew on the host population, and the formation of a mating pair was determined as follows. Each male was ranked by the phenotypic value of *Y*, and males succeeded in mating only in the top *μ* proportion. Each female was randomly assigned one male among the successful males as the father of her offspring. To reflect a broad ecological aspect of mating skew, no specific mechanism of sexual selection was assumed in the simulation, and I assumed that the fixed parameter *μ* scales the strength of sexual selection or the degree of mating skew.

Next, the degree of mating skew that could evolve under the different correlation *ρ* was examined, and comparisons were made of the evolution of mating skew between cases of male–male competition and female mate choice. For these purposes, a second continuous trait, *m*, was used which determined individual efforts in mating behavior (mating trait). In cases of male–male competition, a host male randomly attempted to mate with a certain number of females (the number was denoted by *M*), and for each female, the male with the highest *Y* was the successful mate. In this simulation, the value of mating trait *m* was modeled to a range from 0.0 to 1.0 and determined the number of mating attempts as *M *= *mN*_*H*_. The higher the value of *m* in the population, the less likely that poor‐quality males would find a mate. For the female mate choice, a host female randomly screened *M* males by the male *Y* phenotype value and mated with the male with the highest *Y* value. The number of males screened was determined by the value of her mating trait*, m*, in the same way as for the male–male competition. Therefore, for both cases of sexual selection, the mean value of the host mating trait strongly associated with the degree of mating skew in the host population: Mating success should be more biased toward the males with the higher male phenotype value, as the mean value of mating trait increases in the population.

### Simulation analysis of the individual‐based model

After the mating interaction, each host female produced 20 offspring (The number of offspring was determined for simplicity of model analysis, and I have confirmed that the difference in offspring number did not affect the results of preliminary simulations). Host offspring inherited relevant alleles from both parents with equal probability and free recombination due to sexual reproduction, and offspring sex was determined randomly. For the parasite reproduction, each parasite female also produced 20 offspring. However, because of asexual reproduction, the offspring were all female and clonally inherited the alleles from their mother, except for mutations. Mutations occurred at the time of offspring production. Alleles of the male continuous *y* trait were added by mutation displacement based on a normal distribution with a mean of 0.0 and standard deviation of 0.01. Mutations between alleles 0 and 1 at immunity and infectivity loci occurred mutually at a rate of 1.0 × 10^−5^. In the second simulation, alleles of the mating trait *m* were also added by mutational displacement in the same way as the mutation of the male trait *y*.

Each simulation ran for 2000 generations and iterated 50 times for each parameter set. Two and eight parasite generations per single host generation were used in all simulation runs (these generation times were determined arbitrarily to simplify the simulation analysis, especially for eight generation times, and I confirmed that the results were qualitatively robust for 6–12 parasite generation times in preliminary simulations). The mean rate of infection in the host population and the frequency of recognition loci (host immunity and parasite infectivity) were recorded. In the first simulation, to compare the effects of mating skew and the correlation between male phenotype and state of infection, the value of the degree of mating skew *μ* varied from 0.0 to 1.0, and the value of the correlation *ρ* varied from 0.0 to 1.0. In the second simulation, the mean value of the mating trait *m* in the host population, and the correlation *ρ* varied from 0.0 to 1.0, and this was recorded to examine the effect of the correlation on the evolution of the mating trait. If not otherwise specified, all other conditions were as follows: host and parasite population sizes were *N̂*_*H*_ = *N̂*_*P*_ = 500; the scale of selection by parasite infection was *θ*_*H*_ = 0.25; the scale of selection by host resistance *θ*_*P*_ = 0.75; the value of male trait at the initial condition was 0.0; the value of mating trait at the initial condition was *m *=* *0.01.

## Results

### Effects of mating skew under different degrees of correlation between immunity and male trait

**Figure 1:**
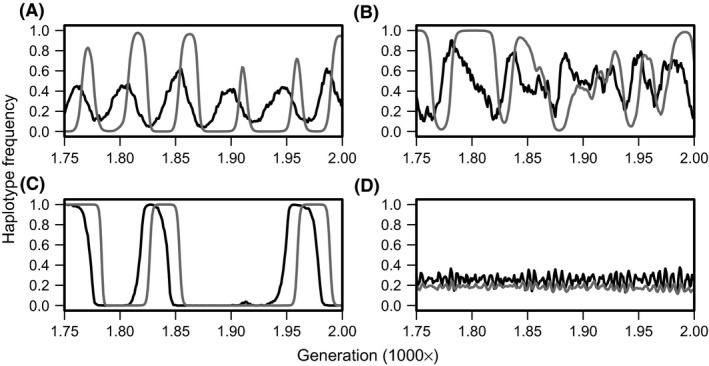
Example dynamics of haplotype 00 in the host population and the parasite population. Black and gray lines represent the haplotype dynamics of host immunity and parasite infectivity, respectively. A degree of mating skew and a value of correlation are different between the panels (A: *μ *= 0.00, *ρ *= 0.00; B: *μ *= 0.90, *ρ *= 0.00; C: *μ *= 0.25, *ρ *= 0.50; D: *μ *= 0.60, *ρ *= 0.50). Other parameter used here: two parasite generations, *θ*_*H*_ = 0.25, *θ*_*P*_ = 0.75.

Figure 1 shows a typical example of the dynamics of a corresponding haplotype for host immunity and parasite infectivity. The host immunity and the parasite infectivity were under frequency‐dependent selection and periodically oscillated in cases of random mating (i.e., *μ *= *ρ *= 0.0; Fig. 1A). However, in cases of mating skew toward male phenotype, the haplotype dynamics of host immunity fluctuated more intensely in cases of high mating skew without genetic correlation (Fig. 1B) than in a random mating system. This could be because strong genetic drift decreased the genetic diversity of host immunity. Moreover, the coevolution between the host and parasite took on a new dynamic when there was a genetic correlation between male trait and male condition. The genetic variation of the host immunity decreased considerably in cases of a moderate mating skew (Fig. 1C), whereas it was maintained in cases of a higher mating skew (Fig. 1D).

**Figure 2:**
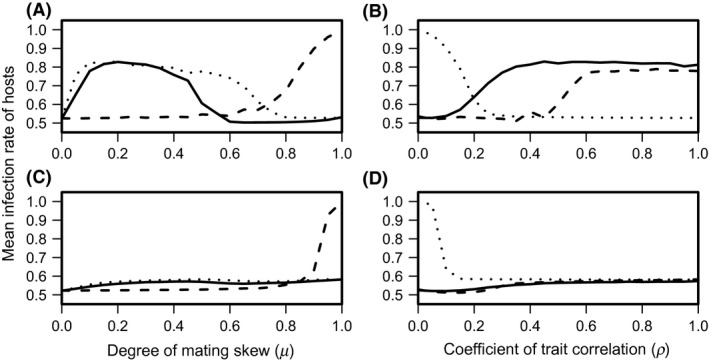
Mean infection rate in the host population over 50 simulations. The panels A and C represent the results of the host infection rate as a function of the degree of mating skew on the host infection (solid lines: *ρ *= 0.50; dashed lines: *ρ *= 0.00; dotted lines: *ρ *= 1.00). The panels B and D represent the results of the host infection rate as a function of the correlation (solid lines: *μ *= 0.25; dashed lines: *μ *= 0.50; dotted lines: *μ *= 1.00). The top panels are the results of the case of two parasite generations, and the bottom panels are the results of the case of eight parasite generations. Other parameters used here are same as Figure 1.

These results indicated that mating skew and the genetic correlation between male trait and male condition did indeed affect genetic variation in the hosts, compared to the results of a random mating system. To investigate the effect of mating skew on the infection rate of hosts, the simulation was performed with varying degrees of mating skew and different correlations. As shown in Figure 2, the results differed between the long and short parasite generation times. First, without a substantial correlation between male trait and male condition, the host population suffered from more serious infections under a higher degree of mating skew (*μ *≥ 0.8) for both parasite generation times (dashed lines in Fig. 2A and C). However, when the male trait was correlated with health, the host infection rate was considerably higher in a moderate degree of mating skew (0.0 < *μ *< 0.5) in longer parasite generation times (Fig. 2A), whereas the host infection rate slightly increased as a function of the mating skew in the short parasite generation times (Fig. 2C). Thus, the effect of correlation on the host infection rate changed depending on the degree of mating skew and the parasite generation. Under the highest mating skew (*μ *= 1.0), the infection rate decreased as the genetic correlation between the male trait and male condition strengthened (dotted lines in Fig. 2B and D). By contrast, the infection rate increased as a function of the correlation under a moderate degree of mating skew, but disease pathology was more serious under longer parasite generation times (Fig. 2B) than shorter parasite generation times (Fig. 2D).

### Evolution of mating skew in the case of male–male competition and female mate choice

**Figure 3:**
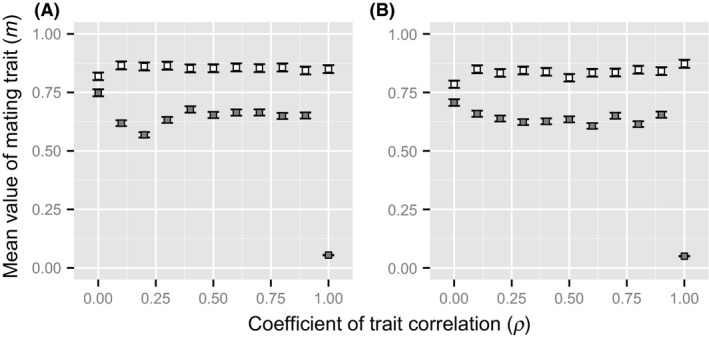
Effects of the difference in the correlation on the evolution of the mating skew. Open squares and filled squared represent the mean values of mating trait in the case of male–male competition and the case of female mate choice, respectively. Error bar is standard deviation over 50 simulation runs. The parameter of parasite generations used in the simulation is different between the two panels (A: 2 generations; B: 8 generations). Other parameters are same as Figure 2.

The results of first simulation suggest that mating skew could worsen infections in the host population because it changed the coevolutionary relationship between hosts and parasites. The second simulation was carried out to investigate how strong mating skews were able to evolve, even with the cost of intensified parasite infection. Figure 3 shows the simulation results of the evolution of the host mating trait in the case of male–male competition (open square) and in those of female mate choice (filled square). In male–male competition, the difference in the correlation had a small effect on the evolution of the mating trait under both parasite generation timescales; however, the mating trait evolved to a high value compared to its initial value (i.e., *m *=* *0.01), regardless of the value of correlation. On the other hand, in the case of female mate choice, an increase in the correlation decreased the value of the mating trait under both parasite generation timescales. However, the mating trait evolved to be higher than its initial value except in the case of the highest value of the correlation (i.e., *ρ *= 1.0). Additionally, a higher mating trait was more likely to evolve in cases of male–male competition than in cases of female mate choice, but the difference in parasite generation timescales had little effect on the evolution of the mating trait.

**Figure 4:**
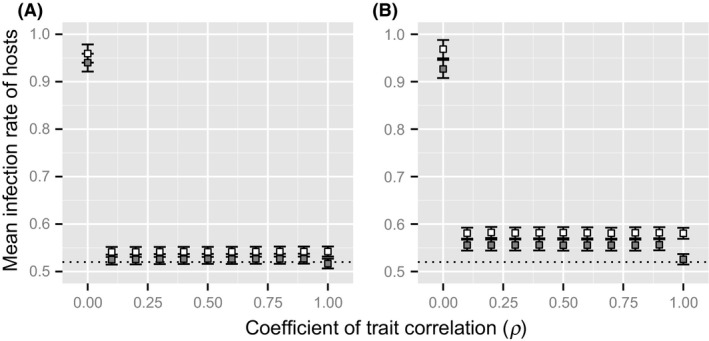
Effects of the difference in the correlation on parasite load in the host. Open and filled squared represent the mean infection rate in the host population in the case of male–male competition and the case of female mate choice, respectively. Dotted line represents the mean infection rate in the host population under random mating. Error bar indicates standard deviation over 50 simulation runs. The parameter of parasite generations used in the simulation is different between the two panels (A: 2 generations, B: 8 generations). Other parameters are same as Figure 2.

In the simulation, the evolution of the mating trait induced changes in the host–parasite interaction. As shown in Figure 4, an increase in the correlation decreased the mean infection rates of the host population in the case of sexual selection, regardless of parasite generation timescales. Specifically, in the case of male–male competition, the mean infection rate was highest when *ρ *= 0.0 compared to the other results for both parasite generation timescales. Moreover, the host population in the male–male competition system suffered more serious infections compared to the random mating system (represented by the dotted lines) for all the correlations in the long‐ and short‐term parasite generations. On the other hand, in the case of female mate choice, the mean infection rate was highest when *ρ *= 0.0 and lowest when *ρ *= 1.0 compared the results of the others for both timescales of parasite generations. The host population of female mate choice also suffered from more infections than those from the random mating system for all the correlations, other than *ρ *= 1.0 in the long‐ and short‐term generations. The mean infection rate of the host population was also higher in cases of male–male competition than in female mate choice, and it differed among the parasite generations times; the differences in the results of sexual selection and the random mating system were smaller for long parasite generation times (Fig. 4A) than those for short parasite generation times (Fig. 4B).

## Discussion

In summary, the IBM simulation analyses revealed that mating skew affected the infection rate of the host population. Specifically, an increase in the degree of mating skew frequently intensified an infection load on the hosts when there was some genetic correlation between a male trait and male immunity against parasites in the sexually selected phenotype. This was because the strengthened mating skews disrupted the cycle of the host–parasite interactions compared to that under a random mating system, which enabled the parasites to shorten the lag between host and parasite frequency dynamics (Fig. 1C). Even when the sexually selected phenotype indicated no male immunity, an increase in the degree of mating skew strengthened the effect of genetic drift under a finite population size, and markedly decreased genetic variation of the host immunity (Fig. 1B). Therefore, the infection rate of the host population increased with an increase in mating skew (Fig. 2). However, the effect of mating skew on the infection rate of the hosts may differ among parasite generation times. Specifically, for long parasite generation times, the haplotype diversity of hosts was maintained in the case of higher mating bias (Fig. 1D) compared to lower mating bias (Fig. 1C). As a result, an extreme mating bias seemed to improve the infection rate of the hosts compared to random mating systems (Fig. 2A). This may occur for the following reasons. First, mating bias for the sexual trait correlated with male immunity should increase the rate at which the host immunity evolves. Second, parasites with long generation times should have a relatively slow evolutionary rate. Therefore, although a moderate mating bias intensified the infection rate, host populations under an extreme mating bias would have a rate higher than that at which infection by parasites with long generation times evolves, which results in improving the infection rate of the hosts.

This model showed that the effect of mating skew and correlation also depends on parasite generation times. This result seems to be inconsistent with that of previous models – parasite generation time affects the period of oscillation, whereas the amplitude of the oscillation is mainly driven by the strength of selection pressure (Lively 1999; Gandon 2002). This inconsistency should be explained by the following. Unlike these previous simulations, additive selection pressure for host immunity, that is, sexual selection or mating skew was incorporated into the model. Therefore, the degree of mating skew affects the host infection rate (Fig. 2A and C), which implies that the amplitude of the oscillations depends on the strength of sexual selection. In addition, because sexual selection only acts in the host population, parasites with shorter generation times should rapidly adapt to the host immunity, which weakens the effect of sexual selection in the host.

The results imply that an increase in mating skew may impose a cost of increased infection on the host population. Nevertheless, analysis of the second simulation demonstrated that when the sexually selected phenotype conveyed some information of male immunity, mating skew evolved in cases of male–male competition and female mate choice (Fig. 3). As a result, the host population actually suffered an increased parasite infection compared with a random mating system (Fig. 4). In addition, although the host infection rate seemed to be higher in the results from short‐term generations (Fig. 4B) than those from long‐term generations (Fig. 4A), there was no difference in the evolution of mating skew between parasite generation timescales. The results indicated that given that the mating skew conferred a reproductive benefit on males and females, it evolved easily, even with the added cost of an increased parasite infection. In fact, when only the haplotype of the male immunity determined the sexually selected phenotype (i.e., the correlation *ρ *= 1.0), no mating skews evolved in the case of female mate choice (Fig. 4). This occurred because mating with high immunity ensured little immunity for the next generation offspring due to the higher speed of counter‐adaptation of the parasites. Moreover, preference for males with high immunity conferred no indirect reproductive benefit through sons of females because other genetic qualities did not affect male reproductive success, which was different when there was genetic association between the male trait and male immunity. These considerations support the inference made above, in which the risk of parasite infection had little effect on the evolution of mating skew when sexual selection for other traits existed. In fact, the simulation analysis demonstrated that the genetic correlation between the male trait and male immunity had little effect on the evolution of mating skew among hosts (Fig. 3). Thus, the results indicated that when any genetic quality other than male immunity affected the sexually selected phenotype, sexual selection on the genetic quality of the male contributed to the evolution of mating skew and affected the parasite infection among the hosts.

The analyses of the IBMs also provided a new understanding of the relationship between the RQH and sexual selection. The results from the second model demonstrated that mating skew evolved more easily in male–male competition than in female mate choice (Fig. 3). This could be because mating skew evolved by direct selection in male–male competition: Males with high‐quality sexually selected phenotypes gained high reproductive success when they realized a strong degree of mating skew. On the other hand, the indirect reproductive benefit gained through sons with a high genetic quality is a major factor that promotes the evolution of mating skew among females in the case of female mate choice. The strength of indirect selection is mathematically calculated using the strength of direct selection weighted with the genetic correlation between the traits (i.e., the male trait and the mating trait in the IBMs): Because the degree of genetic correlation does not exceed 1, indirect selection should be weaker than direct selection (Gavrilets et al. 2001; Cameron et al. 2003). Thus, when male–male competition plays an important role in sexual selection, it is predicted that relatively high mating skews will evolve; hence, the risk of parasite infection increases in the host population. To test this prediction, it would be useful to compare the parasite load between closely related species where the main type of sexual selection differs between male–male competition and female mate choice. For example, for many birds birdsong serves multiple functions; in sexual selection, it plays a role in male–male competition (territory and mate defense) rather than simply being a means by which females evaluate the quality of the males (Garamaszegi 2005). However, few studies have investigated the relationship between the mating skew and the risk of parasite infection among closely related bird species. Further studies are needed to understand the effects of differences in the type of sexual selection on the evolution of mating skew and parasite load.

The evidence based on the simulation analyses revealed that when considering sexual selection and the resultant evolution of mating skew, the effects of the RQH on hosts should depend on its mating system. This perspective will aid our understanding of conditions in which the RQH favors sexual reproduction in real organisms. Many empirical studies have confirmed the RQH and benefits of sexual reproduction of hosts under host–parasite coevolution. These studies involved diverse host–parasite systems, including a freshwater snail and a parasitic trematode (Lively 1987; Lively and Dybdahl 2000), psychid moths and hymenopteran parasitoids (Kumpulainen et al. 2004), and topminnow and helminth (Lively et al. 1990). The host species used in these studies should have various mating systems. To the best of my knowledge, however, little work has been done on the relationship between the RQH and host mating system. Therefore, further studies that compare the effects of the RQH under various host mating systems are required to understand the relationship between the maintenance of sexual reproduction and the RQH.

## Conflict of Interest

None declared.
